# Untargeted Mutation Triggered by Ribonucleoside Embedded in DNA

**DOI:** 10.3390/ijms252413708

**Published:** 2024-12-22

**Authors:** Tetsuya Suzuki, Kiyoharu Yasui, Yasuo Komatsu, Hiroyuki Kamiya

**Affiliations:** 1 Graduate School of Biomedical and Health Sciences, Hiroshima University, 1-2-3 Kasumi, Minami-ku, Hiroshima 734-8553, Japan; suzukite@hiroshima-u.ac.jp (T.S.);; 2 Department of Life Science and Biotechnology, National Institute of Advanced Industrial Science and Technology (AIST), 1-1-1 Umezono, Tsukuba, Ibaraki 305-8560, Japan; komatsu-yasuo@aist.go.jp

**Keywords:** ribonucleoside, action-at-a-distance mutation, APOBEC3

## Abstract

DNA polymerases frequently misincorporate ribonucleoside 5′-triphosphates into nascent DNA strands. This study examined the effects of an incorporated ribonucleoside on untargeted mutations in human cells. Riboguanosine (rG) was introduced into the downstream region of the *supF* gene to preferentially detect the untargeted mutations. The plasmid containing rG was transfected into U2OS cells and the replicated DNA was recovered after 48 h. The mutation analysis using the indicator *Escherichia coli* RF01 strain showed the frequent induction of untargeted base substitutions at the G bases of 5′-GpA-3′ dinucleotides, similar to action-at-a-distance mutations induced by an oxidatively damaged base, 8-oxo-7,8-dihydroguanine, and an apolipoprotein B mRNA-editing enzyme, catalytic polypeptide-like 3 (APOBEC3) cytosine deaminase. APOBEC3B was then knocked down by RNA interference and the plasmid bearing rG was introduced into the knockdown cells. The untargeted mutations at 5′-*G*pA-3′ sites were reduced by ~80%. These results suggested that ribonucleosides embedded in DNA induce base substitution mutations at G bases in the same strand by an APOBEC3B-dependent mechanism, implying that ribonucleosides contribute to APOBEC3-dependent cancer initiation events.

## 1. Introduction

Ribonucleoside 5′-triphosphates are RNA precursors, and their concentrations in mammalian cells are 10- to 100-fold higher than those of 2′-deoxyribonucleoside 5′-triphosphates [1]. Ribonucleoside diphosphate reductase catalyzes the radical reduction reactions from ribonucleoside 5′-diphosphates to the cognate 2′-deoxyribonucleoside 5′-diphosphates [2]. The 2′-deoxyribonucleoside 5′-diphosphates are phosphorylated by nucleoside diphosphate kinase to form the DNA precursor triphosphates [3]. The ribonucleotides and 2′-deoxyribonucleotides differ in terms of the presence/absence of the 2′-hydroxy group. DNA polymerases (pols) must discriminate rNTPs and dNTPs, and the steric gate amino acid residues in their active sites suppress the misincorporation of rNTPs [4]. However, human DNA pol δ incorporates rNTPs at frequencies of 0.05–0.1% in vitro [5]. Likewise, the dNTP/rNTP selectivity is ~10^2^ for the exonuclease-deficient N-terminal polymerization domain of *Saccharomyces cerevisiae* DNA pol ε [6]. In the presence of physiologically relevant dNTP/rNTP concentrations, the calculated discrimination factors of human DNA pol ε (wild type) are 210 (dTTP/rUTP), 300 (dGTP/rGTP and dCTP/rCTP), and 5300 (dATP/rATP) [7]. Human DNA pol β, the repair DNA pol, incorporates rNTPs 10,000 times less efficiently than dNTPs [8]. Meanwhile, human DNA pol γ, the mitochondrial DNA pol, efficiently inserts rNTPs [9]. Moreover, the genomic DNA of mouse embryos lacking RNase H2, an important enzyme preventing ribonucleotide accumulation in DNA (see below), contains over 1 × 10^6^ ribonucleotides per cell [10]. Thus, ribonucleotides embedded within DNA can elicit drastic problems if they inhibit replication and/or induce mutations.

The mammalian RNase H2 protein recognizes ribonucleotides in DNA and catalyzes the hydrolysis of their 5′-phosphodiester bonds, leaving 3′-hydroxy groups and 5′-ribonucleoside-phosphates. Single ribonucleotides in DNA are substrates for RNase H2, in contrast to RNase H1, which requires four contiguous ribonucleotides. The embryonic lethality of the RNase H2 knockout indicates the relevance of this ribonucleotide excision repair (RER) enzyme [10,11]. In addition, the fact that loss-of-function mutations in one of the three genes encoding RNase H2 subunits cause Aicardi–Goutières syndrome, a disease particularly affecting the brain and skin [12], also highlights its importance. Moreover, the gene encoding the RNase H2B subunit is frequently deleted in metastatic prostate cancer and chronic lymphocytic leukemia [13]. Thus, the RER pathway involving RNase H2 is the major pathway serving as a defense against the ribonucleotides incorporated into DNA. Based on biochemical reactions with enzymes purified from yeast, RER is considered to proceed as follows: cleavage of the DNA backbone by RNase H2, strand displacement synthesis by DNA pol δ, flap removal by Flap endonuclease 1 (FEN1), and nick ligation by DNA ligase I [14].

Another ribonucleotide processing pathway involves removal by topoisomerase 1 (TOP1) [15,16]. The TOP1 pathway is considered to back up the repair by RNase H2. TOP1 cleaves the 3′-phosphodiester bond of the ribonucleotide, producing a 5′-hydroxy group and a TOP1 cleavage complex (TOP1cc), the crosslinked TOP1 tyrosine residue and the 3′-phosphate. The 2′-hydroxy group of the ribonucleotide attacks the phosphate atom of the complex, leaving the free TOP1 and a 2′,3′-cyclic phosphate group at the nicked 3′-terminus. The second cleavage of the upstream region of the nick by TOP1 (the second TOP1cc formation) and subsequent TOP1 proteolysis, DNA end processing, DNA synthesis, and sealing by DNA ligase I complete the error-free DNA repair. The tyrosyl-DNA phosphodiesterases TDP1 and TDP2 may remove the phosphotyrosyl bond after the second TOP1 proteolysis [17,18,19,20].

Sassa et al. examined the mutagenic consequences of riboguanosine (rG) embedded in plasmid DNA in human TSCER2 cells (note that the nucleoside, but not the nucleotide, name is used for simplicity) [21,22]. The predominant mutations were large deletions, with most >100 bp. Moreover, the large deletions were suppressed by disrupting the *TDP1* and *TDP2* genes. Another type of mutation induced by rG was untargeted base substitutions, and their induction was independent of TDP1 and TDP2.

Recently, we reported that 8-oxo-7,8-dihydro-2′-deoxyguanosine (dG^O^, also known as 8-hydroxy-2′-deoxyguanosine), one of the major products of DNA oxidation, causes untargeted base substitution mutations at the G bases of 5′-GpA-3′ dinucleotides (the C bases of 5′-TpC-3′), in addition to the well-known targeted G:C→T:A mutation, in human U2OS cells [23]. These action-at-a-distance mutations involve OGG1, the major DNA glycosylase for dG^O^ (OGG1 paradox), and apolipoprotein B mRNA-editing enzyme, catalytic polypeptide-like 3 (APOBEC3)B, which deaminates C bases preferentially at 5′-TpC-3′ sites [24,25]. The current mechanistic hypothesis includes nick formation by apurinic/apyrimidinic endonuclease 1 (APE1) after OGG1 removes the oxidized base to produce an apurinic/apyrimidinic (abasic) site, digestion of the nicked DNA strand, and APOBEC3B-catalyzed deamination reactions on C bases in the exposed single-stranded (ss) DNA (according to the report by Chen et al.) [26]. The nicked DNA produced by APE1 has 3′-hydroxy and 5′-deoxyribose 5-phosphate moieties.

We noticed that nicked DNA is the common product of the OGG1-APE1 (dG^O^) and RNase H2 (ribonucleoside) reactions, although the terminal structures are non-identical. Thus, the ribonucleosides, as dG^O^, may induce action-at-a-distance mutations. In this study, we examined whether rG embedded in DNA causes untargeted mutations in human cells and whether APOBEC3 is involved in the mutagenic process. These hypotheses were supported by the results described in this paper. Thus, the rNTP incorporation into DNA damages the genome integrity in various ways. This genomic instability could cause various genetic diseases when it occurs in germ-line cells, as well as cancers when it occurs in somatic cells.

## 2. Results

### 2.1. Highly Frequent Mutation Induction by rG in DNA

First, we examined whether rG embedded in DNA induces action-at-a-distance mutations in U2OS cells. The rG was placed 14 nucleotides downstream of the *supF* gene, a commonly used mutational reporter gene. This position is named “position 176” for convenience, although it is outside of the coding region (the length of the gene is 162 bp) [23]. This plasmid DNA was prepared by annealing a 5′-phosphorylated oligodeoxyribonucleotide (ODN) containing a single rG residue (Table 1) to the ss form of DNA, subsequent DNA pol plus ligase reactions, and adenine methylation to confer the bacterial methylation pattern [27].

**Figure 1:**
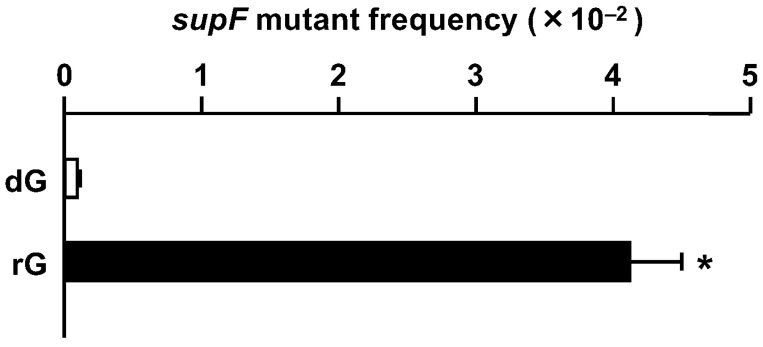
Induction of *supF* mutants by rG. The control (dG-) and rG-plasmid DNAs were introduced into U2OS cells by lipofection. The indicator *E. coli* RF01 was transformed by the plasmid DNAs amplified in the cells. The *supF* mutants were selected by nalidixic acid and streptomycin in the selection plates. The *supF* mutant frequencies were calculated as the ratios of the antibiotic-resistant colonies. Transfection experiments were performed three times. Data are expressed as the means + standard errors. * *p* < 0.05 vs. dG.

The rG-plasmid or the control dG-plasmid was introduced into U2OS cells by lipofection, using Lipofectamine 2000. The replicated DNA was recovered from the transfected cells after 48 h and treated with *Dpn* I to digest unreplicated DNA. The indicator *Escherichia coli* RF01 cells were then transformed by the plasmid DNA [28]. As shown in Figure 1, rG induced *supF* mutants with a frequency of 4.1 × 10^−2^, ~50-fold higher than that of dG (8.2 × 10^−4^). Thus, rG located outside the *supF* gene strongly induced mutations within the gene.

The number of colonies on the titer plates semi-quantitatively reflects the replication efficiency in U2OS cells. The number for the rG-plasmid was approximately half of that for the control plasmid, suggesting partial replication inhibition by the presence of the ribonucleoside (Supplementary Table S1).

### 2.2. G Bases of 5′-GpA-3′ as Mutated Positions

Next, we analyzed the sequences of the *supF*-containing region. In contrast to TSCER2 cells, large deletions were infrequent in U2OS cells (Table 2 and Supplementary Table S2) [21,22]. Base substitution mutations occurred at G:C pairs, mostly at the G bases of 5′-GpA-3′ dinucleotides, in the presence of rG (Table 3 and Table 4). These substitutions were also observed when dG^O^ was incorporated into plasmid DNA [23,25]. Moreover, like dG^O^, these mutations were found as “clusters” for rG, indicating that the same kind of mutation was generated by the rG and dG^O^ nucleosides (Supplementary Table S2 and Figure S1).

**Figure 2:**
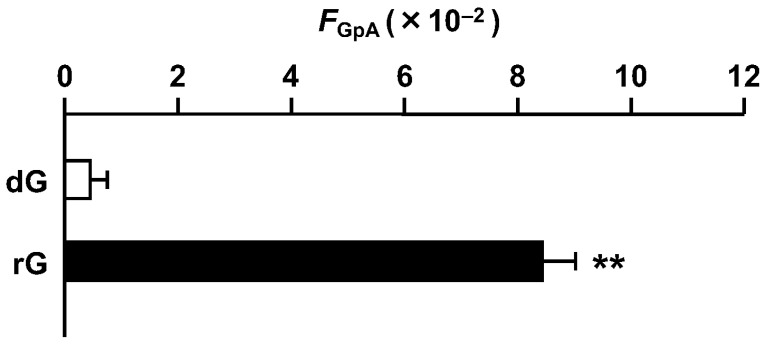
Effect of rG on the frequency of substitution mutations at 5′-GpA-3′ dinucleotides (*F*_GpA_ value). The value was calculated as the product of the *supF* mutant frequency, the ratio of colonies containing any base substitution mutation(s), and the ratio of the total number of 5′-*G*pA-3′ mutations, as described in the text. Data are expressed as means + standard errors. ** *p* < 0.01 vs. dG.

We calculated the *F*_GpA_ value, the product of the *supF* mutant frequency, the ratio of colonies containing any base substitution mutation(s) to the total colonies analyzed (allowing the barcode duplication), and the ratio of the total number of 5′-*G*pA-3′ mutations to the colonies containing any base substitution mutation(s) (excluding the barcode duplication) [23]. The value for the rG-plasmid was 8.4 × 10^−2^, ~50-fold higher than that of the control plasmid (4.3 × 10^−4^) (Figure 2). The substantial increase in base substitution mutations at the G bases of 5′-GpA-3′ strongly suggested that 3 is involved in the mutagenesis process.

### 2.3. Effects of APOBEC3B Knockdown

**Figure 3:**
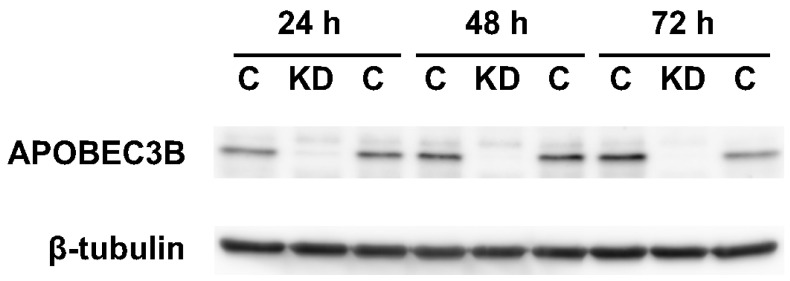
Knockdown of APOBEC3B by siRNA. The levels of APOBEC3B expression in U2OS cells at 24, 48, and 72 h after siRNA introduction, detected by Western blot analysis. ImageJ v.1.53c software was used for the quantitation [30]. C, cells treated with the control RNA; KD, knockdown cells.

In U2OS cells, APOBEC3B is the major molecule among the seven human APOBEC3 cytosine deaminases [25,29]. We knocked down APOBEC3B with siRNA against the protein (Table 1) and examined the effects on the action-at-a-distance mutations. The siRNA was introduced into U2OS cells by lipofection, using Lipofectamine RNAiMAX. The knockdown efficiencies were 62, 84, and 78% at the protein level after 24, 48, and 72 h, respectively (n = 2) (Figure 3).

The rG-plasmid DNA was transfected by Lipofectamine 2000 at 24 h after siRNA introduction. The replicated DNA was extracted from the cells, treated with *Dpn* I, and electroporated into RF01. The numbers of colonies relative to that for dG in the control cells were 59, 9.6, and 6.3% for dG in the knockdown cells, rG in the control cells, and rG in the knockdown cells, respectively (Supplementary Table S3). Thus, the ABOPEC3B knockdown and rG inhibited replication.

**Figure 4:**
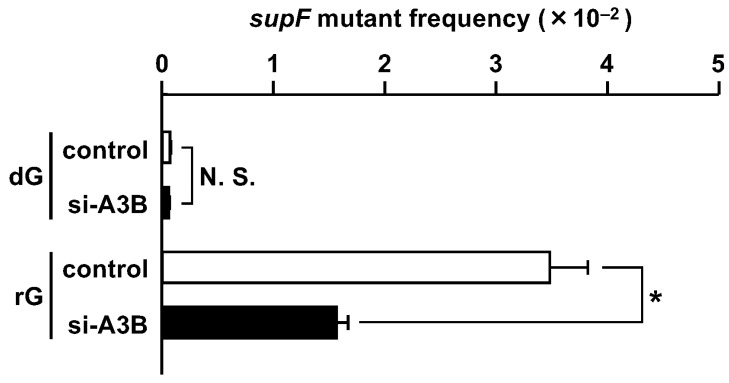
Effects of the APOBEC3B knockdown on the *supF* mutant frequency induced by rG. APOBEC3B was knocked down using si-APOBEC3B, and then the control plasmid or the rG-plasmid was introduced into the knockdown cells. The *supF* mutant frequency was calculated as described in Figure 1. Transfection experiments were performed three times. Data are expressed as the means + standard errors; control, control RNA; si-A3B, si-APOBEC3B; N. S., not significant. * *p* < 0.05.

As shown in Figure 4, the *supF* mutant frequency was significantly reduced by ~55% in the APOBEC3B-knockdown cells. Subsequent mutation analysis indicated that base substitution mutations at G:C pairs were remarkably decreased (Table 5 and Table 6 and Supplementary Tables S4 and S5) by the knockdown. The substitutions at G bases of 5′-GpA-3′ dinucleotides were predominant in the knockdown cells (Table 7). The rG induced mutations in the *supF* gene much more robustly than dG in the knockdown cells, although the total mutant frequency was decreased.

**Figure 5:**
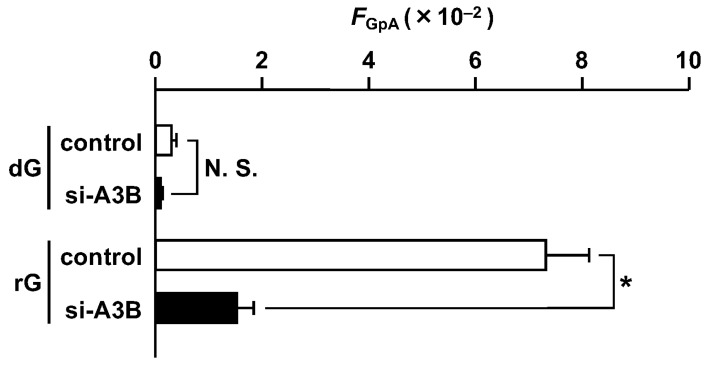
Effects of the APOBEC3B knockdown on the frequency of substitution mutations at 5′-GpA-3′ dinucleotides (*F*_GpA_ value). The value was calculated as described in Figure 2. Data are expressed as the means + standard errors; control, control RNA; si-A3B, si-APOBEC3B; N. S., not significant. * *p* < 0.05.

The effect of APOBEC3B reduction is clearly shown by the *F*_GpA_ values (Figure 5). The substitution frequency at the G bases of 5′-GpA-3′ dinucleotides for rG was lowered to 20% compared to the control RNA-treated cells. Therefore, most of the untargeted substitutions induced by rG were due to the APOBEC3B cytosine deaminase in U2OS cells.

## 3. Discussion

**Figure 6:**
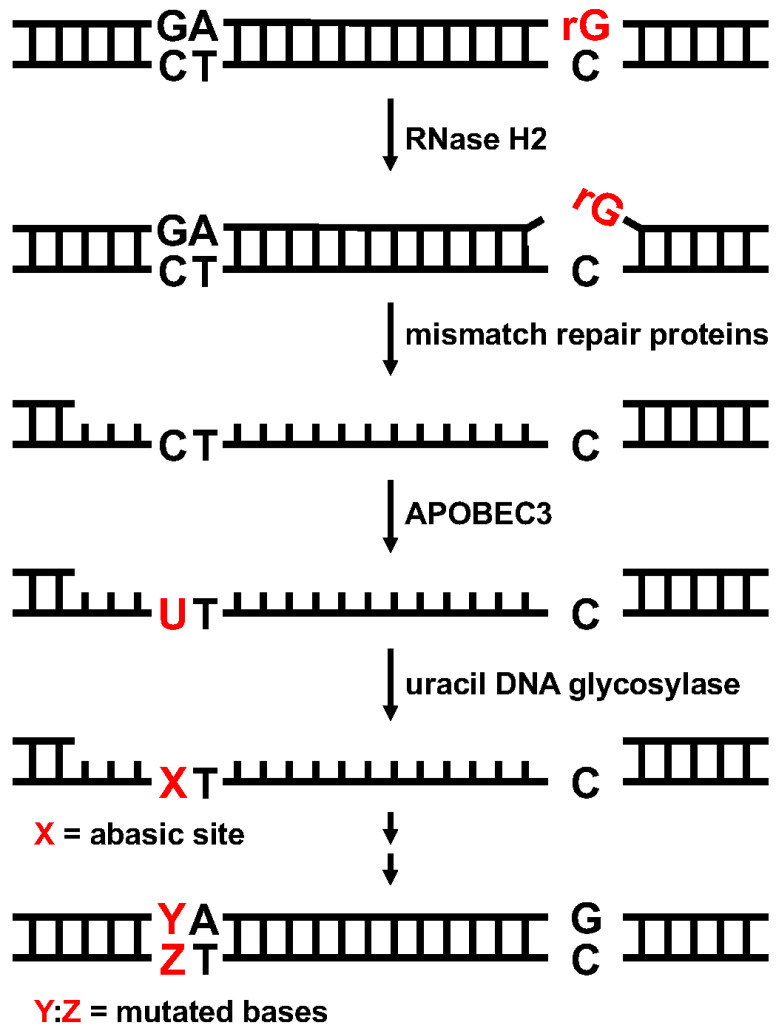
Hypothesized mechanism of APOBEC3-dependent mutagenesis triggered by ribonucleoside.

This study revealed that the rG residue embedded within DNA induces action-at-a-distance mutations at 5′-*G*pA-3′ (5′-Tp*C*-3′) in human U2OS cells, and that this type of mutation is dependent on the cytosine deaminase APOBEC3. As described in the Introduction section, we previously reported that APOBEC3 is involved in similar mutations induced by the oxidatively damaged nucleoside (base) dG^O^ [25]. These mutations were possibly triggered by nick formation by APE1 (dG^O^) or RNase H2 (rG), and the subsequent processes might be conducted by common proteins (for rG, see Figure 6). However, the mutation inducibilities of dG^O^ and rG were quite different. The *F*_GpA_ values of dG^O^ and rG were ~6 × 10^−3^ [27] and ~8 × 10^−2^ (Figure 2), respectively, when both dG^O^ and rG were placed at the same site (position 176). In the case of dG^O^, the DNA glycosylase OGG1 removes the oxidized G base and APE1 then incises the strand to generate 3′-hydroxy plus 5′-deoxyribose-phosphate termini. In contrast, RNase H2 directly nicks the strand to produce 3′-hydroxy plus 5′-ribonucleoside-phosphate termini. The nick formation efficiency may influence the untargeted mutation frequencies.

As described above, RER is considered to be initiated by RNase H2-mediated cleavage of the DNA backbone, followed by strand displacement synthesis by DNA pol δ, flap removal by FEN1, and nick ligation by DNA ligase I [14]. In contrast, the major repair pathway of dG^O^ is short-patch BER, in which DNA pol β fills a one-nucleotide gap [31,32]. The distinct repair intermediate (nicked DNA) structures could lead to different repair efficiencies. Alternatively, the non-identical terminal structures of the nicked DNA might affect the recruitment of the putative protein(s) that initiates the mutagenic process. In addition to the nicking efficiency by OGG1 plus APE1 or RNase H2, the ratio of incorrect (mutagenic) to correct (repair) pathways would determine the untargeted mutation frequency, resulting in the higher mutation frequency of rG.

The nick formation and subsequent strand degradation could result in the exposure of the ss region in the strand complementary to the rG strand in the transfected DNA, based on the report by Chen et al. [26]. Mismatch repair proteins are hypothesized to be involved in this strand degradation (Figure 6). The APOBEC3 enzymes deaminate C bases on ss DNA more efficiently than those on ds DNA [33]. The involvement of APOBEC3 in action-at-a-distance mutations was indicated by our previous and present studies (dG^O^ and rG, respectively) [25]. The formed U bases would be removed by uracil DNA glycosylase to produce abasic (apyrimidinic) sites, and this non-instructive lesion would cause various substitution mutations [34,35]. Specialized translesion synthesis DNA pol(s) are expected to bypass the abasic sites during DNA synthesis where the exposed ss DNA acts as the template.

We compared the distributions of untargeted substitutions for rG, dG^O^, and an abasic site analog (tetrahydrofuran-like analog, THF) (Supplementary Figure S1) [27]. THF is an unnatural “nucleoside” that induces action-at-a-distance mutations (the *F*_GpA_ value is ~5 × 10^−2^). The overall distributions were similar and the major hot spots were positions 5, 27, 91, and 126 of the *supF* gene. As discussed previously [35], various factors including the DNA secondary structure and the biochemical properties of APOBEC3B seem to affect the formation of these hot spots.

As shown in Figure 4 and Figure 5, the APOBEC3B knockdown decreased the mutations by rG and particularly the base substitutions at 5′-*G*pA-3′ sites. The knockdown efficiency was 62% at 24 h after siRNA induction, and the *F*_GpA_ value was reduced to one-fifth of that in the control cells. Likewise, in the case of dG^O^, the knockdown efficiency at the protein level was 74% at 24 h after siRNA treatment, and the *F*_GpA_ value was decreased to a quarter compared to the control cells [25]. Although the absolute mutation frequencies were completely different, the APOBEC3B knockdown had similar impacts on the substitutions at 5′-*G*pA-3′ sites.

Previously, Sassa’s research group, including two of us (TS and HK), reported that rG induces untargeted base substitutions in TSCER2 cells derived from human lymphoblastoid TK6 cells [22]. We reanalyzed the mutation spectrum data in the literature, focusing on the base substitutions at the G bases of 5′-GpA-3′ dinucleotides. Eleven among the sixty colonies analyzed contained mutations at the sites. Since no barcode was used in the study, a direct, quantitative comparison is impossible. However, the results clearly indicated the occurrence of substitutions at 5′-*G*pA-3′ in the presence of rG in TSCER2 cells, as found in the U2OS cells used in this study, suggesting the general nature of this mutagenic phenomenon.

Most of the substitutions at 5′-*G*pA-3′ sequences triggered by rG were dependent on APOBEC3B, as well as dG^O^, in U2OS cells (Figure 5) [25]. Recently, the involvement of APOBEC3A, rather than APOBEC3B, was suggested by mutation signature analyses in various tumors [36,37,38]. Notably, our findings are not limited to events in U2OS cells, since similar untargeted base substitution mutations triggered by T:G and U:G pairs are dependent on APOBEC3 enzymes, especially APOBEC3B, in HeLa cells [26]. In addition, connections between APOBEC3B upregulation by infection with human papillomavirus/BK polyomavirus and tumorigenesis have been reported [39,40,41,42,43,44]. However, the expression of APOBEC3A is detected in myeloid lineage cells but not many other cultured cell lines, and the expression of exogenous APOBEC3A is toxic in HEK293 cells [45,46], indicating that experiments focusing on APOBEC3A are challenging in cultured cells. The results shown in this study provide the general conclusion that ribonucleotide incorporation into DNA could induce substitution mutations at various sites in human cells in which at least one member of the APOBEC3 cytosine deaminases is active against DNA.

When plasmid DNAs amplified in U2OS cells were introduced into indicator *E. coli* cells, the number of colonies on the titer agar plates was much lower (approximately half) for the rG group than for the dG group. Thus, the presence of a single ribonucleoside seemed to inhibit the plasmid replication. In vitro, yeast DNA pols δ and ε bypass rA with ~70% efficiencies of those of dA [47]. The relative bypass efficiencies of rA and rG by human DNA pol δ are ~80% [5]. Moreover, rG does not retard DNA synthesis by human DNA pols α and η, while the bypass by human DNA pol κ is poor [48]. In addition, some rG strands of plasmid DNAs should be cleaved by RNase H2 in the U2OS cells and replication might thus be inhibited. The reduction of colony numbers on the titer plates would reflect the suppression of replication by both rG and the nicked structure.

In summary, rG embedded in DNA induced untargeted base substitution mutations at the G bases of 5′-GpA-3′ dinucleotides, and the APOBEC3B knockdown robustly reduced these mutations. The properties of the induced mutations were similar to those of dG^O^, suggesting that the same proteins involving APOBEC3 drive this mutagenesis event. This study revealed that the presence of the 2′-hydroxy group in the sugar had a remarkably negative influence on the genome integrity.

## 4. Materials and Methods

### 4.1. Materials

The 5′-phosphorylated ODN containing rG was synthesized by GeneDesign (Ibaraki, Osaka, Japan) and purified by HPLC (the procedures are described in detail in Supplementary Methods). The control ODN with dG was synthesized and purified by Hokkaido System Science (Sapporo, Japan). The DsiRNA against APOBEC3B (si-APOBEC3B) and the nontargeting DsiRNA (DS NC1, control RNA) were purchased from Integrated DNA Technologies (Coralville, IA, USA). The sequences of the ODNs and DsiRNAs are shown in Table 1.

### 4.2. Cell Line and Cell Culture

The human osteosarcoma U2OS cells were purchased from American Type Culture Collection (Manassas, VA, USA, ATCC HTB-96) and were cultured in Dulbecco’s modified Eagle’s medium (Nissui Pharmaceutical, Tokyo, Japan), supplemented with 10% (*v*/*v*) fetal bovine serum and 1× Antibiotic-Antimycotic Mixed Stock Solution (Nacalai Tesque, Kyoto, Japan), at 37 °C in a humidified atmosphere with 5% CO_2_.

### 4.3. Plasmid DNA Construction

The double-stranded pSB189KL-BC (D12) plasmids with dG or rG were constructed from the ss form of DNA and the 5′-phosphorylated dG or rG ODN (Table 1), as described previously [27,49]. The procedures are described in detail in the Supplementary Methods.

### 4.4. siRNA and Plasmid Transfections and supF Mutation Analyses

Transfections of siRNAs and plasmids into U2OS cells were performed as described previously [24,25]. Briefly, 2.5 nM siRNA was reverse transfected into U2OS cells (1.0 × 10^5^ cells/well in 12-well plates) with 0.25 μL Lipofectamine RNAiMAX (Thermo Fisher Scientific, Waltham, MA, USA). No siRNA transfection was performed in experiments without a knockdown. After 24 h, 200 ng of the plasmid (59 fmol) was introduced with 0.6 μL of Lipofectamine 2000 (Thermo Fisher Scientific). After 48 h, the plasmid was extracted from the cells and unreplicated plasmid was digested with *Dpn* I, as described previously [23]. The replicated plasmid was introduced into the indicator *E. coli* RF01 strain to calculate the *supF* mutant frequency [28]. The mutation spectra of the *supF* gene were analyzed by sequencing the plasmids obtained from the colonies on the selection plates. The procedures are described in detail in the Supplementary Methods.

### 4.5. Western Blotting

At 24, 48, and 72 h after siRNA transfection, cells were lysed by radioimmunoprecipitation (RIPA) buffer to obtain whole cell extracts. Western blot analysis was performed as described previously [25,30]. The procedures are described in detail in the Supplementary Methods and Supplementary Table S6.

### 4.6. Statistics

Statistical significance for the *supF* mutant frequencies or the *F*_GpA_ values was examined with Welch’s *t*-test. For statistical analysis of mutation spectra, the Fisher’s exact test was used with the Bonferroni correction for multiple comparisons. To examine which specific types of mutations were significantly different between dG and rG groups or control and si-APOBEC3B groups, the Fisher’s exact test was performed separately for 2 × 2 sub-tables for each type of mutation. Each sub-table consisted of a number of the mutation type of interest and the total number of all other mutations in each group. The level of statistical significance was set at less than 0.05 for *p* values.

## Figures and Tables

**Table 1:** Oligonucleotides used in this study.

Oligonucleotide	Sequence (5′ → 3′)
ODNs for plasmid DNA construction	
ODN-1 ^a^	P-GATCCGGCGGCGCAGCACCAT
ODN-2 ^a,b^	P-GATCCGGCG**rG**CGCAGCACCAT
	
	
siRNAs	
si-APOBEC3B sense ^c^	ACCUACGAUGAGUUUGAGUACUGCT
si-APOBEC3B antisense	AGCAGUACUCAAACUCAUCGUAGGUCA
DS NC1 sense ^c^	CGUUAAUCGCGUAUAAUACGCGUAT
DS NC1 antisense	AUACGCGUAUUAUACGCGAUUAACGAC

^a^ P represents the phosphate. ^b^ rG is shown in bold. ^c^ Underline indicates DNA.

**Table 2:** Overall mutation spectra ^a,b^.

		dG		rG	
untargeted substitution					
	at A:T pair	20	(24)	6	(7)
	at G:C pair	92	(108)	200	(235)
targeted substitution		N.A. ^c^		1	(1)
small insertion (1–2 bp)		0	(0)	2	(2)
large insertion (>2 bp)		1	(1)	1	(1)
small deletion (1–2 bp)		0	(0)	5	(6)
large deletion (>2 bp)		11	(13)	9	(11)
rearrangement or complex		0	(0)	1	(1)
unknown		4	(5)	6	(7)
				
total mutations		128		231	
total colonies analyzed		85	(100)	85	(100)

^a^ All data are represented as cases found (%). ^b^ Statistically significant between dG and rG (*p* < 0.001, Fisher’s exact test). ^c^ Not applicable.

**Table 3:** Untargeted base substitution mutations ^a,b^.

		dG		rG	
transition					
	A:T → G:C	3	(8)	0	(0)
	G:C → A:T	10	(28)	75	(41)
				
transversion					
	A:T → T:A	0	(0)	5	(3)
	A:T → C:G	3	(8)	0	(0)
	G:C → T:A	7	(19)	40	(22)
	G:C → C:G	13	(36)	62	(34)
				
total base substitutions		36	(100)	182	(100)

^a^ All data are represented as cases found (%). Barcode-identical colonies are excluded. ^b^ Statistically significant between dG and rG (*p* < 0.001, Fisher’s exact test).

**Table 4:** Dinucleotide signatures of mutations ^a^.

		dG		rG	
C mutations					
	AC	0	(0)	0	(0)
	TC	14	(47)	1	(1)
	GC	0	(0)	0	(0)
	CC	2	(7)	1	(1)
total C mutations		16	(53)	2	(1)
				
G mutations					
	GA	12	(40)	154	(87)
	GT	1	(3)	4	(2)
	GG	1	(3)	13	(7)
	GC	0	(0)	5	(3)
total G mutations		14	(47)	176	(99)
				
total base substitutions at G:C sites		30	(100)	178	(100)

^a^ The sequence of the upper strand is shown. The percentages are shown in parentheses.

**Table 5:** Overall mutation spectra ^a^.

		dG				rG			
		Control		si-APOBEC3B ^b^		Control		si-APOBEC3B ^c^	
untargeted substitution									
	at A:T pair	2	(3)	11	(18)	3	(5)	6	(10)
	at G:C pair	55	(92)	45	(75)	156	(260)	73	(122)
targeted substitution		N.A. ^d^		N.A. ^d^		0	(0)	0	(0)
small insertion (1–2 bp)		1	(2)	0	(0)	0	(0)	0	(0)
large insertion (>2 bp)		0	(0)	0	(0)	0	(0)	0	(0)
small deletion (1–2 bp)		3	(5)	3	(5)	5	(8)	0	(0)
large deletion (>2 bp)		16	(27)	6	(10)	9	(15)	15	(25)
rearrangement or complex		1	(2)	2	(3)	2	(3)	7	(12)
unknown		1	(2)	2	(3)	1	(2)	2	(3)
								
total mutations		79		69		176		103	
total colonies analyzed		60	(100)	60	(100)	60	(100)	60	(100)

^a^ All data are represented as cases found (%). ^b^ Statistically significant versus control, dG (*p* < 0.05, Fisher’s exact test). ^c^ Statistically significant versus control, rG (*p* < 0.001, Fisher’s exact test). ^d^ Not applicable.

**Table 6:** Untargeted base substitution mutations ^a^.

		dG				rG			
		Control		si-APOBEC3B		Control		si-APOBEC3B	
transition									
	A:T → G:C	2	(4)	3	(9)	0	(0)	0	(0)
	G:C → A:T	13	(28)	7	(21)	65	(42)	20	(29)
								
transversion									
	A:T → T:A	0	(0)	1	(3)	3	(2)	4	(6)
	A:T → C:G	0	(0)	1	(3)	0	(0)	1	(1)
	G:C → T:A	9	(20)	8	(24)	34	(22)	14	(21)
	G:C → C:G	22	(48)	13	(39)	53	(34)	29	(43)
									
total base substitutions		46	(100)	33	(100)	155	(100)	68	(100)

^a^ All data are represented as cases found (%). Barcode-identical colonies are excluded.

**Table 7:** Dinucleotide signatures of mutations at G and C ^a^.

		dG				rG			
		Control		si-APOBEC3B		Control		si-APOBEC3B	
C mutations									
	AC	0	(0)	0	(0)	0	(0)	0	(0)
	TC	18	(41)	11	(39)	1	(1)	1	(2)
	GC	0	(0)	1	(4)	1	(1)	0	(0)
	CC	1	(2)	3	(11)	5	(3)	1	(2)
total C mutations		19	(43)	15	(54)	7	(5)	2	(3)
								
G mutations									
	GA	21	(48)	7	(25)	124	(82)	51	(81)
	GT	1	(2)	0	(0)	4	(3)	2	(3)
	GG	3	(7)	6	(21)	13	(9)	9	(14)
	GC	0	(0)	0	(0)	4	(3)	0	(0)
total G mutations		25	(57)	13	(46)	145	(95)	61	(97)
								
total base substitutions at G:C sites		44	(100)	28	(100)	152	(100)	63	(100)

^a^ The sequence of the upper strand is shown. The percentages are shown in parentheses.

## Data Availability

Source data for Figure 3 are provided with the paper. The data that support the findings of this study are available from the corresponding author upon reasonable request.

## Supplementary Materials

The supporting information can be downloaded at: https://www.mdpi.com/article/10.3390/ijms252413708/s1.

## Abbreviations

APOBEC3, apolipoprotein B mRNA-editing enzyme, catalytic polypeptide-like 3; dG^O^, 8-oxo-7,8-dihydro-2′-deoxyguanosine; ODN, oligodeoxyribonucleotide; pol, polymerase; RER, ribonucleotide excision repair; rG, riboguanosine; ss, single stranded; THF; tetrahydrofuran-like analog; TOP1, topoisomerase 1.

## References

[1] Traut T.W. (1994). Physiological concentrations of purines and pyrimidines. Mol. Cell. Biochem..

[2] Jordan A., Reichard P. (1998). Ribonucleotide reductases. Ann. Rev. Biochem..

[3] Boissan M., Schlattner U., Lacombe M.L. (2018). The NDPK/NME superfamily: State of the art. Lab. Investig..

[4] Joyce C.M. (1997). Choosing the right sugar: How polymerases select a nucleotide substrate. Proc. Natl. Acad. Sci. USA.

[5] Clausen A.R., Zhang S., Burgers P.M., Lee M.Y., Kunkel T.A. (2013). Ribonucleotide incorporation, proofreading and bypass by human DNA polymerase δ. DNA Repair..

[6] Nick McElhinny S.A., Kumar D., Clark A.B., Watt D.L., Watts B.E., Lundström E.B., Johansson E., Chabes A., Kunkel T.A. (2010). Genome instability due to ribonucleotide incorporation into DNA. Nat. Chem. Biol..

[7] Göksenin A.Y., Zahurancik W., LeCompte K.G., Taggart D.J., Suo Z., Pursell Z.F. (2012). Human DNA polymerase is able to efficiently extend from multiple consecutive ribonucleotides. J. Biol. Chem..

[8] Cavanaugh N.A., Beard W.A., Wilson S.H. (2010). DNA polymerase ribonucleotide discrimination: Insertion, misinsertion, extension, and coding. J. Biol. Chem..

[9] Kasiviswanathan R., Copeland W.C. (2011). Ribonucleotide discrimination and reverse transcription by the human mitochondrial DNA polymerase. J. Biol. Chem..

[10] Reijns M.A.M., Rabe B., Rigby R.E., Mill P., Astell K.R., Lettice L.A., Boyle S., Leitch A., Keighren M., Kilanowski F. (2012). Enzymatic removal of ribonucleotides from DNA is essential for mammalian genome integrity and development. Cell.

[11] Hiller B., Achleitner M., Glage S., Naumann R., Behrendt R., Roers A. (2012). Mammalian RNase H2 removes ribonucleotides from DNA to maintain genome integrity. J. Exp. Med..

[12] Crow Y.J., Leitch A., Hayward B.E., Garner A., Parmar R., Griffith E., Ali M., Semple C., Aicardi J., Babul-Hirji R. (2006). Mutations in genes encoding ribonuclease H2 subunits cause Aicardi-Goutières syndrome and mimic congenital viral brain infection. Nat. Genet..

[13] Zimmermann M., Murina O., Reijns M.A., Agathanggelou A., Challis R., Tarnauskaitė Ž., Muir M., Fluteau A., Aregger M., McEwan A. (2018). CRISPR screens identify genomic ribonucleotides as a source of PARP-trapping lesions. Nature.

[14] Sparks J.L., Chon H., Cerritelli S.M., Kunkel T.A., Johansson E., Crouch R.J., Burgers P.M. (2012). RNase H2-initiated ribonucleotide excision repair. Mol. Cell.

[15] Sekiguchi J., Shuman S. (1997). Site-specific ribonuclease activity of eukaryotic DNA topoisomerase I. Mol. Cell.

[16] Kim N., Huang S.N., Williams J.S., Li Y.C., Clark A.B., Cho J.E., Kunkel T.A., Pommier Y., Jinks-Robertson S. (2011). Mutagenic processing of ribonucleotides in DNA by yeast topoisomerase I. Science.

[17] Yang S.W., Burgin A.B., Huizenga B.N., Robertson C.A., Yao K.C., Nash H.A. (1996). A eukaryotic enzyme that can disjoin dead-end covalent complexes between DNA and type I topoisomerases. Proc. Natl. Acad. Sci. USA.

[18] Interthal H., Chen H.J., Champoux J.J. (2005). Human Tdp1 cleaves a broad spectrum of substrates, including phosphoamide linkages. J. Biol. Chem..

[19] Zeng Z., Sharma A., Ju L., Murai J., Umans L., Vermeire L., Pommier Y., Takeda S., Huylebroeck D., Caldecott K.W. (2012). TDP2 promotes repair of topoisomerase Imediated DNA damage in the absence of TDP1. Nucleic Acids Res..

[20] Tsuda M., Kitamasu K., Kumagai C., Sugiyama K., Nakano T., Ide H. (2020). Tyrosyl-DNA phosphodiesterase 2 (TDP2) repairs topoisomerase 1 DNA-protein crosslinks and 3′-blocking lesions in the absence of tyrosyl-DNA phosphodiesterase 1 (TDP1). DNA Repair..

[21] Sassa A., Tada H., Takeishi A., Harada K., Suzuki M., Tsuda M., Sasanuma H., Takeda S., Sugasawa K., Yasui M. (2019). Processing of a single ribonucleotide embedded into DNA by human nucleotide excision repair and DNA polymerase η. Sci. Rep..

[22] Takeishi A., Kogashi H., Odagiri M., Sasanuma H., Takeda S., Yasui M., Honma M., Suzuki T., Kamiya H., Sugasawa K. (2020). Tyrosyl-DNA phosphodiesterases are involved in mutagenic events at a ribonucleotide embedded into DNA in human cells. PLoS ONE.

[23] Suzuki T., Masuda H., Mori M., Ito R., Kamiya H. (2021). Action-at-a-distance mutations at 5′-GpA-3′ sites induced by oxidised guanine in WRN-knockdown cells. Mutagenesis.

[24] Suzuki T., Zaima Y., Fujikawa Y., Fukushima R., Kamiya H. (2022). Paradoxical role of the major DNA repair protein, OGG1, in action-at-a-distance mutation induction by 8-oxo-7,8-dihydroguanine. DNA Repair..

[25] Fukushima R., Suzuki T., Kobayakawa A., Kamiya H. (2024). Action-at-a-distance mutations induced by 8-oxo-7,8-dihydroguanine are dependent on APOBEC3. Mutagenesis.

[26] Chen J., Miller B.F., Furano A.V. (2014). Repair of naturally occurring mismatches can induce mutations in flanking DNA. eLife.

[27] Fukushima R., Suzuki T., Komatsu Y., Kamiya H. (2022). Biased distribution of action-at-a-distance mutations by 8-oxo-7,8-dihydroguanine. Mutat. Res./Fundam. Mol. Mech. Mutagen..

[28] Fukushima R., Suzuki T., Kamiya H. (2020). New indicator *Escherichia coli* strain for rapid and accurate detection of supF mutations. Genes Environ..

[29] Constantin D., Dubuis G., Conde-Rubio M.D., Widmann C. (2022). APOBEC3C, a nucleolar protein induced by genotoxins, is excluded from DNA damage sites. FEBS J..

[30] Schneider C.A., Rasband W.S., Eliceiri K.W. (2012). NIH Image to ImageJ: 25 years of image analysis. Nat. Methods.

[31] Barnes D.E., Lindahl T. (2004). Repair and genetic consequences of endogenous DNA base damage in mammalian cells. Annu. Rev. Genet..

[32] Fortini P., Parlanti E., Sidorkina O.M., Laval J., Dogliotti E. (1999). The type of DNA glycosylase determines the base excision repair pathway in mammalian cells. J. Biol. Chem..

[33] Pecori R., Di Giorgio S., Lorenzo J.P., Papavasiliou F.N. (2022). Functions and consequences of AID/APOBEC-mediated DNA and RNA deamination. Nat. Rev. Genet..

[34] Gentil A., Cabral-Neto J.B., Mariage-Samson R., Margot A., Imbach J.L., Rayner B., Sarasin A. (1992). Mutagenicity of a unique apurinic/apyrimidinic site in mammalian cells. J. Mol. Biol..

[35] Suzuki T., Yoshida S., Kamiya H. (2024). Inhibition of uracil DNA glycosylase alters frequency and spectrum of action-at-a-distance mutations induced by 8-oxo-7,8-dihydroguanine. Biol. Pharm. Bull..

[36] Buisson R., Langenbucher A., Bowen D., Kwan E.E., Benes C.H., Zou L., Lawrence M.S. (2019). Passenger hotspot mutations in cancer driven by APOBEC3A and mesoscale genomic features. Science.

[37] Cortez L.M., Brown A.L., Dennis M.A., Collins C.D., Brown A.J., Mitchell D., Mertz T.M., Roberts S.A. (2019). APOBEC3A is a prominent cytidine deaminase in breast cancer. PLoS Genet..

[38] Petljak M., Dananberg A., Chu K., Bergstrom E.N., Striepen J., von Morgen P., Chen Y., Shah H., Sale J.E., Alexandrov L.B. (2022). Mechanisms of APOBEC3 mutagenesis in human cancer cells. Nature.

[39] Warren C.J., Xu T., Guo K., Griffin L.M., Westrich J.A., Lee D., Lambert P.F., Santiago M.L., Pyeon D. (2015). APOBEC3A functions as a restriction factor of human papillomavirus. J. Virol..

[40] Vieira V.C., Leonard B., White E.A., Starrett G.J., Temiz N.A., Lorenz L.D., Lee D., Soares M.A., Lambert P.F., Howley P.M. (2014). Human papillomavirus E6 triggers upregulation of the antiviral and cancer genomic DNA deaminase APOBEC3B. mBio.

[41] Henderson S., Chakravarthy A., Su X., Boshoff C., Fenton T.R. (2014). APOBEC-mediated cytosine deamination links PIK3CA helical domain mutations to human papillomavirus-driven tumor development. Cell Rep..

[42] Mori S., Takeuchi T., Ishii Y., Yugawa T., Kiyono T., Nishina H., Kukimoto I. (2017). Human papillomavirus 16 E6 upregulates APOBEC3B via the TEAD transcription factor. J. Virol..

[43] Ohba K., Ichiyama K., Yajima M., Gemma N., Nikaido M., Wu Q., Chong P., Mori S., Yamamoto R., Wong J.E. (2014). In vivo and in vitro studies suggest a possible involvement of HPV infection in the early stage of breast carcinogenesis via APOBEC3B induction. PLoS ONE.

[44] Verhalen B., Starrett G.J., Harris R.S., Jiang M. (2016). Functional upregulation of the DNA cytosine deaminase APOBEC3B by polyomaviruses. J. Virol..

[45] Yamazaki H., Shirakawa K., Matsumoto T., Hirabayashi S., Murakawa Y., Kobayashi M., Sarca A.D., Kazuma Y., Matsui H., Maruyama W. (2019). Endogenous APOBEC3B overexpression constitutively generates DNA substitutions and deletions in myeloma cells. Sci. Rep..

[46] Land A.M., Law E.K., Carpenter M.A., Lackey L., Brown W.L., Harris R.S. (2013). Endogenous APOBEC3A DNA cytosine deaminase is cytoplasmic and nongenotoxic. J. Biol. Chem..

[47] Clausen A.R., Murray M.S., Passer A.R., Pedersen L.C., Kunkel T.A. (2013). Structure-function analysis of ribonucleotide bypass by B family DNA replicases. Proc. Natl. Acad. Sci. USA.

[48] Sassa A., Çağlayan M., Rodriguez Y., Beard W.A., Wilson S.H., Nohmi T., Honma M., Yasui M. (2016). Impact of ribonucleotide backbone on translesion synthesis and repair of 7,8-dihydro-8-oxoguanine. J. Biol. Chem..

[49] Suzuki T., Kamiya H. (2022). Easily-controllable, helper phage-free single-stranded phagemid production system. Genes Environ..

