# Supplementary material for: Untargeted Mutation Triggered by Ribonucleoside Embedded in DNA
Source: Int J Mol Sci. 2024 Dec 22;25(24):13708. doi: 10.3390/ijms252413708 (PMC11679520; doi:10.3390/ijms252413708)
Supplement: Supplementary file 1 [file ijms-25-13708-s001.zip › ijms-3342841-supplementary.v6/Supplmentary_Materials/ijms-3342841-Supplementary methods.pdf]

**Supplementary methods**

*Oligodeoxyribonucleotide containing rG*

The 5'-phosphorylated ODN containing rG was chemically synthesized using standard phosphoramidite chemistry by GeneDesign (Ibaraki, Osaka, Japan). Riboguanosine and terminal phosphate were incorporated by using 5'-dimethoxytrithyl-2'-*O*-TBDMS-amidite unit and chemical phosphorylation reagent (CPR), respectively (Glen Research, Sterling, VA, USA). Cleavage from the solid support and removal of the protecting groups were carried out in 1:1 mixture of 40% methylamine and concentrated ammonia solution at 65°C for 10 min. The deprotection of TBDMS groups was carried out in a solution containing 3HF/triethylamine (75 μL), DMSO (115 μL), and triethylamine (60 μL) at 65°C for 2.5 h. After the reaction, the solution was desalted with an NAP-10 column (Cytiva, Marlborough, MA, USA). The ODN was purified by HPLC using a reversed-phase column, μ-Bondasphere (Waters, Milford, MA, USA). Condition: B buffer contents 0-80% / 20 min (A buffer, 5% acetonitrile-0.1 M triethylamine ammonium (TEAA); B buffer, 25% acetonitrile-0.1 M TEAA).

*Plasmid DNA construction*

*E. coli* HB101 bearing VCSM13CmΔPS ($P_{BAD}$-pII) [27] was transformed by 10 ng of pSB189KL-BC(D12) plasmids and plated on LB agar plates containing 25 μg/mL kanamycin, 30 μg/mL chloramphenicol, and 0.2% glucose. Approximately $10^5$ colonies were harvested and suspended in LB medium containing 15% glycerol at a final $OD_{610}$ of approximately 50. The glycerol stocks were stored at –80°C. A 100 μL portion of the *E. coli* stock was inoculated into 50 mL of 2× YT medium containing 25 μg/mL kanamycin and 30 μg/mL chloramphenicol. The bacteria were cultured at 37°C for 4 h. Arabinose was then added to the culture at a 0.2% final concentration, and the culture was incubated further at 37°C overnight. The phage particles produced from a 50-mL culture was precipitated by adding a one-tenth volume of 20% PEG-6000/2.5 M NaCl solution to the supernatant of the overnight culture. The solution containing the phage precipitate was centrifuged at 12,000 rpm for 15 min at 4°C to concentrate the phage. The pellet was resuspended in 1 mL of 10 mM Tris-HCl (pH 8.0), and then 10 μL of 300 mM $MgCl_2$, 5 units of recombinant DNase I (Takara Bio, Kusatsu, Japan), and 10 mg of RNase A (Nacalai Tesque, Kyoto, Japan) were added, followed by incubation at room temperature for 1 h to remove contaminated bacterial nucleic acids in the supernatant. Afterward, 6.6 μL of 0.5 M EDTA (pH 8.0) was added to the suspension, and then 5 μL of ≥600 mAnson units/mL

proteinase K and 50 µL of 10% SDS were added, and the mixture was incubated at 50°C for 1 h. After this incubation, 0.5 mL of Buffer P3 (Qiagen, Venlo, Netherlands) was added, and the solution was then centrifuged at 12,000 rpm for 5 min at 4°C. The supernatant was applied to a QIAGEN-tip20 column pre-equilibrated with Buffer QBT, and the column was washed with 2 mL of Buffer QC twice. The ss DNA was eluted with 1.6 mL of Buffer QF pre-warmed to 50°C, and then precipitated and resuspended in an appropriate volume of sterile water.

All enzymes used for plasmid construction were purchased from New England BioLabs (Ipswich, MA, USA). Fifteen pmol of the 5′-phosphorylated dG or rG ODN was mixed with 5 µg (3 pmol) of the single-stranded (ss) pSB189KL-BC(D12) in a final volume of 20 µL of 1× Phusion HF Buffer (New England BioLabs). After denaturation at 90°C for 2 min followed by rapid cooling, the solutions were heated at 70°C for 5 min, and then allowed to gradually cool to room temperature (–1°C/min) to hybridize the ODNs with the ss DNAs. Polymerase and ligase reactions of the hybridized DNAs were simultaneously performed in 1× Phusion HF Buffer, supplemented with 1 mM dithiothreitol, 0.2 mM dNTPs, and 1 mM $NAD^+$, using 2 units of Phusion High-Fidelity DNA polymerase and 40 units of *Taq* DNA ligase in a total volume of 100 µL. The sample was incubated at 50°C for 10 min, and then at 65°C for 1 h. After the

sample was vigorously vortexed to inactivate the enzymes, 1 μL of 8 mM *S*-adenosylmethionine, 1 μL of 750 mM $MgCl_2$, 16 units of *dam* methyltransferase, and 10 units of T5 exonuclease were added to the sample. The solution was incubated at 37°C for 3 h to methylate the A bases in the 5′-GATC sequences and to digest the DNAs other than closed circular double-stranded DNA. The DNA was purified with a PureLink PCR Purification Kit (Thermo Fisher Scientific, Waltham, MA, USA) and precipitated with ethanol.

*siRNA and plasmid transfections and supF mutation analyses*

U2OS cells ($1.0\times10^5$ cells/well in 12-well plates) were cultured in 1 mL of Dulbecco's modified Eagle's medium, supplemented with 10% fetal bovine serum. Three pmol of siRNA and 0.25 μL of Lipofectamine RNAiMAX (Thermo Fisher Scientific) were separately diluted in 100 μL of Opti-MEM I (Thermo Fisher Scientific). The diluted siRNA and Lipofectamine RNAiMAX were combined and incubated for 5 min at room temperature. The siRNA-lipid complex was added to the wells immediately after the cells were seeded. No siRNA transfection was performed in experiments without knockdown. After 24 h, the medium was changed to fresh one in the knockdown experiments. Two hundred ng of the plasmids (59 fmol) and 0.6 μL of Lipofectamine 2000 (Thermo Fisher

Scientific) were separately diluted in 50 μL of Opti-MEM I. The diluted plasmid and Lipofectamine 2000 were combined and incubated for 5 min at room temperature. The plasmid-lipid complex was added to the cells and incubated at 37°C for 3 h. The medium was changed to fresh one, and the cells were further cultured at 37°C for 45 h. The cells were collected, and resuspended in 100 μL of TEG (25 mM Tris-HCl, 10 mM EDTA, 50 mM glucose, pH 8.0) solution. The cells were lysed by adding 200 μL of alkali-SDS (0.2 M NaOH and 1% SDS) solution, neutralized by adding 150 μL of 8 M ammonium acetate, and centrifuged at 12,000 rpm for 15 min at 4°C. The plasmid in the supernatant was concentrated by 2-propanol precipitation with 20 μg of glycogen. The precipitated plasmid was dissolved in 15 μL of *Dpn* I reaction solution (1× rCutSmart Buffer, 8 units of *Dpn* I, New England BioLabs), and incubated at 37°C for >2 h to digest unreplicated plasmid. After the reaction, the plasmid was precipitated with ethanol, and dissolved in 10 μL of sterile water.

One μL of the extracted plasmids was electroporated into 40 μL of RF01 competent cells in 0.1-cm gap cuvettes with MicroPulser electroporator (Bio-Rad Laboratories, Hercules, CA, USA) as a following condition: 1.8 kV/cm, 600 Ω, 10 μF. After a 1-h recovery culture in 1 mL of SOC medium, the cultures were seeded on LB titer (25 μg/mL kanamycin and 10 μg/mL chloramphenicol) and

selection (25 μg/mL kanamycin, 10 μg/mL chloramphenicol, 50 μg/mL nalidixic acid, and 100 μg/mL streptomycin) plates. After culturing at 37°C overnight, *supF* mutant frequency was calculated by dividing the number of colonies on the selection plates by that on the titer plates.

*Western blotting*

At 24, 48, and 72 h after siRNA transfection, cells were lysed by radioimmunoprecipitation (RIPA) buffer to obtain whole cell extracts. The extracts were resolved by SDS-12% polyacrylamide gel electrophoresis, and proteins were electroblotted onto PVDF membranes by a wet transfer method. The membranes were subsequently blocked and incubated with the respective primary antibodies. The immunocomplexes were detected with horseradish peroxidase-conjugated secondary antibodies and chemiluminescent reagents. Chemiluminescence signals were scanned with an ImageQuant LAS 4000 mini image analyzer (GE Healthcare, Piscataway, NJ, USA) and, quantified with the ImageJ software [30]. Detailed conditions for western blot are shown in Supplementary Table S6.
