# Supplementary material for: Untargeted Mutation Triggered by Ribonucleoside Embedded in DNA
Source: Int J Mol Sci. 2024 Dec 22;25(24):13708. doi: 10.3390/ijms252413708 (PMC11679520; doi:10.3390/ijms252413708)
Supplement: Supplementary file 1 [file ijms-25-13708-s001.zip › ijms-3342841-supplementary.v6/Supplmentary_Materials/New_Figure S1.pdf]

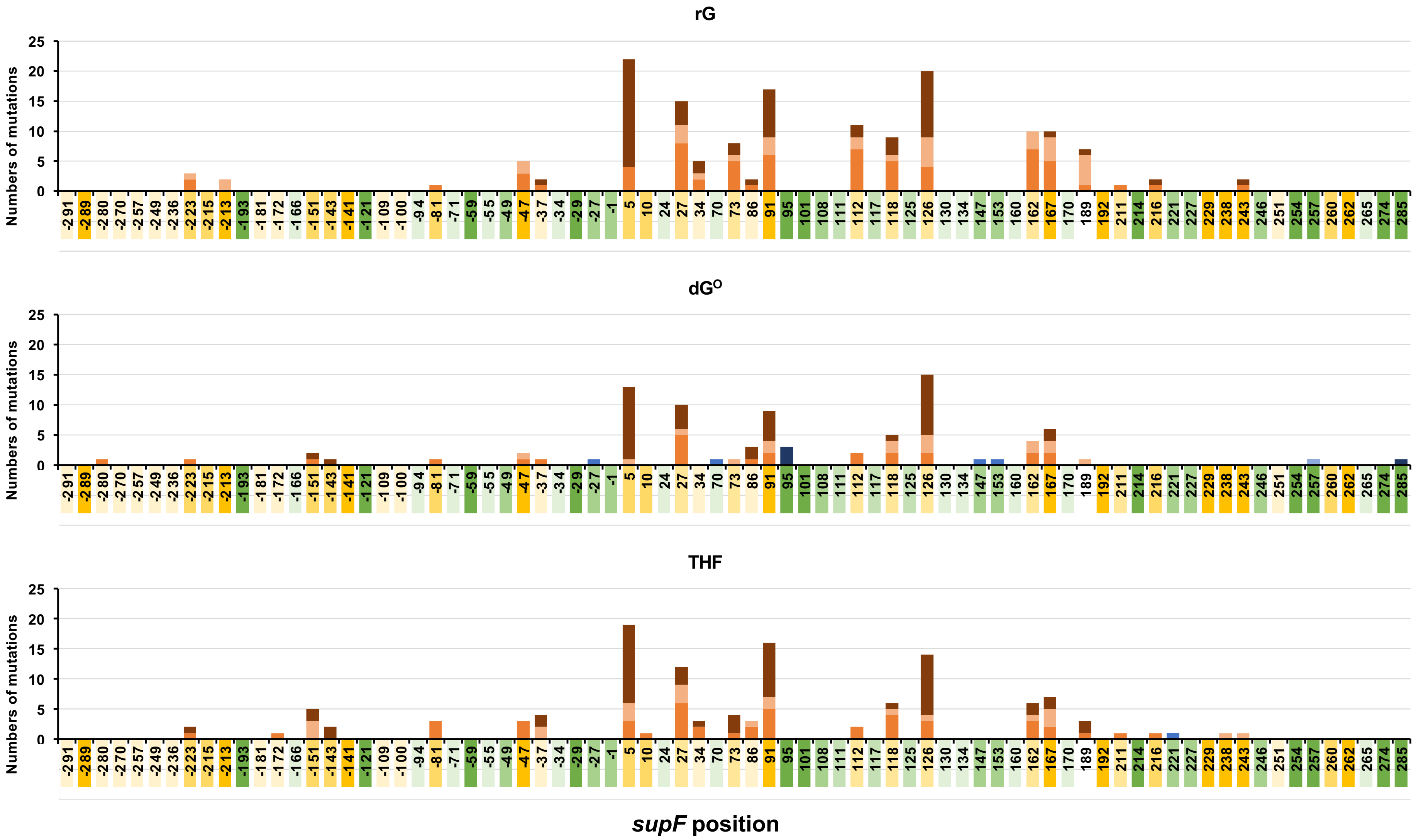

Supplementary Figure S1. Distributions of the base substitution mutations at 5'-GpA-3' and 5'-TpC-3' sequences. Orange, light orange, and dark orange bars indicate G→A, G→T, and G→C mutations, respectively. Blue, light blue, and dark blue bars indicate C→T, C→A, and C→G mutations, respectively. The dG^{O} and THF data are from reference 27.
