# Supplementary material for: Untargeted Mutation Triggered by Ribonucleoside Embedded in DNA
Source: Int J Mol Sci. 2024 Dec 22;25(24):13708. doi: 10.3390/ijms252413708 (PMC11679520; doi:10.3390/ijms252413708)
Supplement: Supplementary file 1 [file ijms-25-13708-s001.zip › ijms-3342841-supplementary.v6/Supplmentary_Materials/Supplementary_TableS1.pdf]

Table S1 The number of colonies after electroporation

		titer plates		selection plates	
		dilution factor	number of colonies	dilution factor	number of colonies
dG	exp. 1	5000	802	5	518
	exp. 2	5000	791	5	1064
	exp. 3	5000	1844	5	854
rG	exp. 1	1000	1985	100	518
	exp. 2	1000	2357	100	1064
	exp. 3	1000	1836	100	854
