# Supplementary material for: Untargeted Mutation Triggered by Ribonucleoside Embedded in DNA
Source: Int J Mol Sci. 2024 Dec 22;25(24):13708. doi: 10.3390/ijms252413708 (PMC11679520; doi:10.3390/ijms252413708)
Supplement: Supplementary file 1 [file ijms-25-13708-s001.zip › ijms-3342841-supplementary.v6/Supplmentary_Materials/Supplementary_TableS2.pdf]

Table S2 Mutations detected in the *supF* gene (dG and rG-plasmids)[a,b]

dG		
–223 G->C, –183 G->C, 91 G->C, 112 G->A	1	(1)
–172 G->A, 126 G->C	1	(1)
–55 C->T, 111 C->A, 130 C->A	1	(1)
5 G->C	4	(2)
5 G->C, 27 G->A	23	(2)
12 T->C	1	(1)
62 G->A	1	(1)
73 G->C, 96 T->G	7	(1)
74 A->C	9	(2)
74 A->G	3	(2)
95 C->G	5	(2)
95 C->G, 111 C->T	3	(2)
101 C->A, 117 C->T	4	(2)
117 C->A	2	(1)
131 C->A	3	(1)
141 C->A	1	(1)
large insertion	1	(1)
large deletion	11	
unknown	4	
total colonies analyzed	85	(24)

rG		
–259 G->A, –182 G->A, 27 G->A, 103 G->C	1	(1)
–223 G->T, 27 G->A, 73 G->A, 91 G->A	1	(1)
–223 G->A, 73 G->A, 112 G->A, 126 G->A	1	(1)
–223 G->A, 118 G->A	1	(1)
–213 G->T, 112 G->A, 118 G->A, 208 ΔA	2	(2)
–129 G->C, 91 G->C, 167 G->T	2	(1)
–81 G->A, –37 G->C, 27 G->C, 73 G->A, 91 G->C, 126 G->A	1	(1)
–47 G->A, 5 G->C, 27 G->A	2	(2)
–47 G->T, 5 G->C, 27 G->A, 73 G->C, 91 G->A, 162 G->T, 189 G->C, 210 C->T	1	(1)
–47 G->T, 27 G->A, 126 G->A, large insertion	1	(1)
–47 G->A, 91 G->T, 112 G->T, 126 G->A, 127 A->T, 189 G->A, 216 G->C, 243 G->C	1	(1)
–44 G->A, 5 G->C, 27 G->T, 67 G->A	1	(1)
–37 G->A, 5 G->C, 243 G->A	1	(1)
5 G->C	6	(5)
5 G->C, 27 G->A	1	(1)
5 G->A, 27 G->A, 34 G->A, 39 G->A, 61 G->A, 65 G->T, 66 G->A, 162 G->T	1	(1)
5 G->C, 27 G->C, 61 G->T, 189 G->T	1	(1)
5 G->A, 34 G->C	1	(1)
5 G->A, 34 G->T, 91 G->C, 112 G->A, 167 G->A, 189 G->T	1	(1)
5 G->C, 65 G->T	1	(1)
5 G->C, 65 G->T, 73 G->T, 106 G->C	1	(1)
5 G->C, 73 G->A, 162 G->A	1	(1)
5 G->A, 91 G->T	1	(1)
5 G->C, 91 G->C, 92 A->T	1	(1)
5 G->C, 167 G->C	1	(1)
27 G->T, 34 G->A, 40 G->C, 103 G->T, 126 ΔG, 163 – 166 AAAA->AAAAA, 167 G->A	1	(1)
27 G->C, 91 G->C, 118 G->T, 210 C->T	1	(1)
27 G->C, 126 G->C, 162 G->A	1	(1)
27 G->T, 126 G->C	1	(1)
34 G->C, 121 G->C	1	(1)
44 G->T, 61 G->A, 73 G->C, 91 G->A	2	(1)
66 G->C, 91 G->A, 162 G->A, 167 G->A, 169 T->A, 170 ΔC	1	(1)
73 G->A, 91 G->T, 126 G->C, 162 G->A, 167 G->T, 169 T->A, 189 G->T	2	(1)
86 G->A, 91 G->A, 112 G->A, 113 A->T, 126 G->T, 162 G->A, 163 – 166 AAAA->AAAAA, 167 G	1	(1)
86 G->C, 176 G->A, 189 G->T	1	(1)
91 G->C, 112 G->T, 126 G->T, 167 G->A	1	(1)
91 G->A, 112 G->A, 162 G->A	1	(1)
91 G->C	2	(2)
112 G->C, 126 G->T	5	(2)
112 G->A, 126 G->C, 158 G->T, 162 G->T	1	(1)
118 G->C, 162 G->A	1	(1)
118 G->A	2	(2)
118 G->C	1	(1)
118 G->C, 167 G->T, 189 G->T	2	(1)
126 G->C	5	(5)
126 G->T	1	(1)
126 G->C, 167 G->T	1	(1)
126 G->C, 183 ΔA	1	(1)
5 G->C, large deletion	1	(1)
large deletion, 216 G->A	1	(1)
large deletion	7	
large deletion + large insertion	1	
unknown	6	
total colonies analyzed	85	(63)

[a]Mutations detected in single colonies are represented. The sequence of the upper strand is shown. The numbers of colonies are shown on the right side. The corrected numbers based on the barcode are shown in parentheses. Positions of the G bases of 5'-GpA-3' and C bases of 5'-TpC-3' are shown in red and blue, respectively.

[b]The original nucleotide sequence is shown in reference 25.
