# Supplementary material for: Untargeted Mutation Triggered by Ribonucleoside Embedded in DNA
Source: Int J Mol Sci. 2024 Dec 22;25(24):13708. doi: 10.3390/ijms252413708 (PMC11679520; doi:10.3390/ijms252413708)
Supplement: Supplementary file 1 [file ijms-25-13708-s001.zip › ijms-3342841-supplementary.v6/Supplmentary_Materials/Supplementary_TableS3.pdf]

Table S3 The number of colonies after electroporation

			titer plates		selection plates	
			dilution factor	number of colonies	dilution factor	number of colonies
dG	control	exp. 1	10000	558	10	454
		exp. 2	5000	940	5	583
		exp. 3	5000	838	5	488
dG	si-APOBEC3B	exp. 1	10000	446	10	226
		exp. 2	2000	406	2	198
		exp. 3	2000	1623	2	949
rG	control	exp. 1	1000	656	100	273
		exp. 2	1000	286	10	902
		exp. 3	1000	453	10	1383
rG	si-APOBEC3B	exp. 1	2000	169	20	292
		exp. 2	500	403	5	652
		exp. 3	1000	365	10	500
