# Supplementary material for: Untargeted Mutation Triggered by Ribonucleoside Embedded in DNA
Source: Int J Mol Sci. 2024 Dec 22;25(24):13708. doi: 10.3390/ijms252413708 (PMC11679520; doi:10.3390/ijms252413708)
Supplement: Supplementary file 1 [file ijms-25-13708-s001.zip › ijms-3342841-supplementary.v6/Supplmentary_Materials/Supplementary_TableS4.pdf]

Table S4 Mutations detected in the *supF* gene (dG-plasmid)[a,b]

control RNA			si-APOBEC3B		
–55 C->T, –29 C->T, 70 C->T, 95 C->G, 111 C->T	1	(1)	–193 C->G, –166 C->G, 101 C->A, 104 C->A	1	(1)
–49 C->T, 70 C->T, 74 A->G	1	(1)	–59 C->A, 12 T->C	1	(1)
–23 G->A, 5 G->C, 27 G->A, 188 ΔA, 243 G->T	1	(1)	–9 ΔC	3	(1)
–9 ΔC	2	(1)	5 G->C	6	(3)
5 G->A	1	(1)	12 T->G	1	(1)
5 G->C	8	(6)	66 G->C	1	(1)
5 G->C, 73 G->A	3	(2)	71 C->G	3	(1)
5 G->C, 138 C->T	1	(1)	74 A->G	5	(1)
66 G->C	1	(1)	94 T->A, 95 C->T	2	(1)
95 C->G	2	(1)	95 C->G	2	(2)
95 C->G, 111 C->A	1	(1)	97 A->G	2	(1)
97 A->G, 108 C->A, 125 C->G	1	(1)	101 C->G, 160 C->G	1	(1)
101 C->A	2	(2)	118 G->A	2	(1)
101 C->G	1	(1)	121 G->A	4	(4)
117 C->A	1	(1)	121 G->T	9	(1)
118 G->C	1	(1)	126 G->C	2	(1)
121 G->A	1	(1)	126 G->C, 189 G->T	1	(1)
121 G->T	1	(1)	130 C->T	2	(1)
126 G->C	7	(4)	130 C->A, 131 C->A	1	(1)
130 C->A	5	(2)	131 C->A	1	(1)
large deletion, 204 +A, 211 G->C	1	(1)	large deletion	6	
large deletion	15		large deletion + large insertion	2	
large deletion + large insertion	1		unknown	2	
unknown	1				
Total analyzed colonies	60	(32)	Total analyzed colonies	60	(26)

[a]Mutations detected in single colonies are represented. The sequence of the upper strand is shown. The numbers of colonies are shown on the right side. The corrected numbers based on the barcode are shown in parentheses. Positions of the G bases of 5'-GpA-3' and C bases of 5'-TpC-3' are shown in red and blue, respectively.

[b]The original nucleotide sequence is shown in reference 25.
