# Supplementary material for: Untargeted Mutation Triggered by Ribonucleoside Embedded in DNA
Source: Int J Mol Sci. 2024 Dec 22;25(24):13708. doi: 10.3390/ijms252413708 (PMC11679520; doi:10.3390/ijms252413708)
Supplement: Supplementary file 1 [file ijms-25-13708-s001.zip › ijms-3342841-supplementary.v6/Supplmentary_Materials/Supplementary_TableS6.pdf]

Table S6 Western blot conditoins

	APOBEC3B	β-tubulin
host, Isotype	rabbit, IgG monoclonal	mouse, IgG1 monoclonal
source/catalog No.	Abcam/ab184990	Wako/014-25041
blocking solution	BlockingOne (Nacalai)	BlockingOne (Nacalai)
primary antibody[a]	1:2500; 1 h at room temperature	1:5000; 1 h at room temperature
secondary antibody[a, b]	1:5000; 1 h at room temperature	1:5000; 1 h at room temperature
chemiluminescent reagent	ImmunoStar LD (Wako)	EzWestLumi Plus (ATTO)
exposure condition sensitivity/time	High; 20 min	Standard; 1 min

[a]Antibodies were diluted in 20-fold diluted BlockingOne in PBS(-) containing 0.05% Tween 20

[b]Horseradish peroxidase-conjugated anti-rabbit and anti-mouse IgGs were obtained from Nacalai (anti-rabbit IgG: 21858-11, anti-mouse IgG: 21860-61).
